# Supplementary material for: Transfer Learning with Kernel Methods
Source: Nat Commun. 2023 Sep 9;14:5570. doi: 10.1038/s41467-023-41215-8 (PMC10492830; doi:10.1038/s41467-023-41215-8)
Supplement: Supplementary file 1 — Supplementary Information [file 41467_2023_41215_MOESM1_ESM.pdf]

Supplementary Materials:
Transfer Learning with Kernel Methods

Adityanarayanan Radhakrishnan[1,2#], Max Ruiz Luyten[1#],
Neha Prasad[1], Caroline Uhler[1,2,*]

[1]Massachusetts Institute of Technology, U.S.A.
[2]Broad Institute of MIT and Harvard, U.S.A.

[#]Equal contribution.
[*]To whom correspondence should be addressed; E-mail: cuhler@mit.edu.

This PDF file includes:

Supplementary Notes 1-5

Supplementary Figures S1 to S9

Supplementary Notes

1 Review of Kernel Regression

We provide a brief review of training kernel machines using kernel ridge regression. For a detailed description of these methods, we refer the reader to [1].

Let $X = [x^{(1)}, \ldots, x^{(n)}] \in \mathbb{R}^{d \times n}$ denote training examples and $y = [y^{(1)}, \ldots, y^{(n)}] \in \mathbb{R}^{1 \times n}$ denote the corresponding labels. The key idea behind kernel machines is to first transform the training examples, X, using a feature map and then perform linear regression on the transformed data to fit the labels, y. In particular, given a feature map $\psi : \mathbb{R}^d \to \mathbb{R}^m$, we can nonlinearly fit the data by solving

$$\arg\min_{w \in \mathbb{R}^m} \|y - w^T \psi(X)\|^2,$$

where $\psi(X) \in \mathbb{R}^{m \times n}$ and the ith column of $\psi(X)$ is $\psi(x^{(i)})$. In cases where m is much larger than d, solving the above system becomes computationally expensive. The key idea behind kernel regression is to assume that w is given by a linear combination of training examples, i.e., $w = \sum_{i=1}^{n} \alpha_i \psi(x^{(i)})$, and then solve the above system for the coefficients $\alpha = [\alpha_1, \ldots \alpha_n] \in \mathbb{R}^{1 \times n}$ instead. Assuming this form for w, the above optimization problem can be written as

$$\arg\min_{\alpha \in \mathbb{R}^n} \sum_{i=1}^{n} \left(y^{(i)} - \alpha \sum_{j=1}^{n} \langle \psi(x^{(j)}), \psi(x^{(i)}) \rangle \right)^2 .$$

Importantly, this optimization problem only depends on the inner product of the feature map between training samples. Thus, instead of working with the feature map directly, we can define a *kernel*, i.e., a positive semi-definite, symmetric function $K : \mathbb{R}^d \times \mathbb{R}^d \to \mathbb{R}$, such that $K(x^{(j)}, x^{(i)}) = \langle \psi(x^{(j)}), \psi(x^{(i)}) \rangle$. The resulting optimization problem is known as *kernel regression* and given as follows:

$$\arg\min_{\alpha \in \mathbb{R}^{1 \times n}} \|y - \alpha K_n\|_2^2 ,$$

where $K_n \in \mathbb{R}^{n \times n}$ with $(K_n)_{i,j} = K(x^{(i)}, x^{(j)})$. Importantly, the abstraction to kernel methods allows using feature maps that map into an infinite dimensional inner product space (i.e., a Hilbert space), which are central to the study of infinite-width neural networks.

In addition to kernel regression described above, we also consider *kernel ridge regression*, which involves modifying the objective with a regularization term with a tunable ridge parameter λ as follows:

$$\arg\min_{\alpha \in \mathbb{R}^{1 \times n}} \|y - \alpha(K_n + \lambda I_{n \times n})\|_2^2 .$$

We primarily use a small non-zero ridge term to avoid numerical issues leading to a singular (or non-invertible) kernel matrix, K_n.

2 Proof of Theorem 1

The proof of Theorem 1 relies on the following lemma.

Lemma 1. Let $D, \Lambda \in \{0, 1\}^{d \times d}$ be two diagonal matrices of rank p and q, respectively. Let $V \in R^{d \times d}$ be an orthogonal matrix and $W \in R^{d \times d}$ a Haar distributed random matrix. If $P = WDW^T, Q = V\Lambda V^T$, then

$$\mathbb{E}_W [PQP] = \frac{p}{d(d-1)(d+2)} \left[q(d-p) I_{d \times d} + [d(p+1) - 2] \, Q \right].$$

Proof. Without loss of generality, assume that $\Lambda = \text{diag}(\mathbf{1}_q, \mathbf{0}_{d-q})$, and $D = \text{diag}(\mathbf{1}_p, \mathbf{0}_{d-p})$. Since the Haar distribution is rotational invariant, $U = V^T W$ is Haar distributed. Therefore,

$$\begin{aligned} \mathbb{E}_W [PQP] &= \mathbb{E}_{W \sim \text{Haar}} \left[WDW^T V \Lambda V^T WDW^T \right] \\ &= V \mathbb{E}_{U \sim \text{Haar}} \left[UDU^T \Lambda UDU^T \right] V^T \\ &= V \mathbb{E}_{U \sim \text{Haar}} \left[A^T A \right] V^T, \end{aligned}$$

where $A = \Lambda U D U^T$. Now the upper left $q \times p$ block of $\Lambda U D$ is equal to the corresponding block in U, and all other entries of $\Lambda U D$ are 0. Letting $\tilde{u}_i = (u_{i1}, \ldots, u_{ip})$, we have

$$A = \begin{pmatrix} \langle \tilde{u}_1, \tilde{u}_1 \rangle & \cdots & \langle \tilde{u}_1, \tilde{u}_d \rangle \\ \vdots & \ddots & \vdots \\ \langle \tilde{u}_q, \tilde{u}_1 \rangle & \cdots & \langle \tilde{u}_q, \tilde{u}_d \rangle \\ 0 & \cdots & 0 \\ \vdots & \ddots & \vdots \\ 0 & \cdots & 0 \end{pmatrix}.$$

Thus, $(A^T A)_{i,r} = \sum_{k=1}^{q} \langle \tilde{u}_i, \tilde{u}_k \rangle \langle \tilde{u}_r, \tilde{u}_k \rangle$, and so, $\mathbb{E}_U[A^T A]$ only depends on the fourth moments of the entries in U. In particular,

$$\mathbb{E}_U \left[(A^T A)_{i,r} \right] = \sum_{\alpha=1}^{q} \sum_{j,s=1}^{p} \mathbb{E}_U \left[u_{ij} u_{rs} u_{\alpha j} u_{\alpha s} \right].$$

To calculate these moments, we use Lemma 9 from [2]. In particular, if $i \neq r$, then $\mathbb{E}_U[(A^T A)_{i,r} = 0$, and if $i = r$, then

$$\mathbb{E}_U \left[u_{ij} u_{is} u_{\alpha j} u_{\alpha s} \right] = \frac{1}{d(d-1)(d+2)} \left[d\delta_{js} + d\delta_{i\alpha} + (d-2)\delta_{i\alpha}\delta_{js} - 1 \right].$$

Therefore, we have the following closed form for the expectation:

$$\mathbb{E}_U \left[(A^T A)_{i,i} \right] = \frac{p}{d(d-1)(d+2)} \left[(d-p) I_{d \times d} + \left[d(p+1) - 2 \right] \Lambda \right].$$

Since $\mathbb{E}_W[PQP] = V \mathbb{E}_U \left[(A^T A) \right] V^T$, $VV^T = I_{d \times d}$, and $V \Lambda V^T = Q$, the result follows.

We now prove the following simpler version of Theorem 1 for the case when $n_s = d$ (i.e., when $\hat{\omega}_s = \omega_s$).

Theorem 3. Let $\mathcal{X} = \mathbb{R}^d, \mathcal{Y}_s = \mathbb{R}^{c_s}, \mathcal{Y}_t = \mathbb{R}^{c_t}$ and let $\hat{\omega}_p = y_t(\omega_s X_t)^\dagger$. Assuming $\mathbb{P}_s, \mathbb{P}_t$ are independent, isotropic distributions on $\mathbb{R}^d$, then

$$\mathcal{R}(\hat{\omega}_p \omega_s) = \left(1 - n_t \frac{d - c_s}{(d+2)(d-1)} \right) \mathcal{R}(\hat{\omega}_t) + n_t \left(\frac{1}{d} + \frac{d - n_t}{(d+2)(d-1)} \right) \varepsilon,$$

where $\varepsilon = ||\omega_t (I_{d \times d} - \omega_s^\dagger \omega_s)|||_F^2$.

Proof. Let $X = X_t$ to simplify notation. Let $\omega_s^{||} = \omega_s^\dagger \omega_s$, $\omega_s^\perp = I_{d \times d} - \omega_s^{||}$, $X^{||} = XX^\dagger$, and $X^\perp = I_{d \times d} - X^{||}$, and note that $\omega_s^\perp, \omega^{||}, X^\perp, X^{||} \in \{0,1\}^{d \times d}$ are all projections. Then, we have:

$$\hat{\omega}_p \omega_s = y_t (\omega_s X)^\dagger \omega_s = \omega_t X X^\dagger \omega_s^\dagger \omega_s = \omega_t X^{||} \omega_s^{||}.$$

Therefore,

$$\begin{aligned} \hat{\omega}_p \omega_s - \omega_t &= \omega_t \left(X^{||} \omega_s^{||} - I_{d \times d} \right) \\ &= \omega_t \left[\left(X^{||} - I_{d \times d} \right) \omega_s^{||} + \omega_s^{||} - I_{d \times d} \right] \\ &= -\omega_t \left[X^\perp \omega_s^{||} + \omega_s^\perp \right]. \end{aligned}$$

Using the cyclic property of the trace, the risk is given by

$$\begin{aligned} \mathcal{R}(\hat{\omega}_p \omega_s) &= \mathbb{E}_{X,x} \left[||\hat{\omega}_p \omega_s x - \omega_t x||^2 \right] \\ &= \operatorname{Tr} \left(\mathbb{E}_{X,x} \left[\omega_t \left(X^\perp \omega_s^{||} + \omega_s^\perp \right) x x^T \left(X^\perp \omega_s^{||} + \omega_s^\perp \right)^T \omega_t^T \right] \right) \\ &= \operatorname{Tr} \left(\mathbb{E}_X \left[\omega_t \left(X^\perp \omega_s^{||} + \omega_s^\perp \right) \left(X^\perp \omega_s^{||} + \omega_s^\perp \right)^T \omega_t^T \right] \right). \end{aligned}$$

Using the idempotent property of projections and the fact that $\omega_s^\perp \omega_s^{||} = \omega_s^{||}\omega_s^\perp = \mathbf{0}$, we conclude that

$$\left(X^\perp \omega_s^{||} + \omega_s^\perp\right)\left(X^\perp \omega_s^{||} + \omega_s^\perp\right)^T = X^\perp \omega_s^{||}\omega_s^{||} X^\perp + X^\perp \omega_s^{||}\omega_s^\perp + \omega_s^\perp \omega_s^{||} X^\perp + \omega_s^\perp \omega_s^\perp$$
$$= X^\perp \omega_s^{||} X^\perp + \omega_s^\perp,$$

and as a consequence that

$$\mathcal{R}(\hat{\omega}_p \omega_s) = \mathrm{Tr}\left(\mathbb{E}_X\left[\omega_t\left(X^\perp \omega_s^{||} X^\perp + \omega_s^\perp\right)\omega_t^T\right]\right)$$
$$= \mathrm{Tr}\left(\omega_t\left(\mathbb{E}_X\left[X^\perp \omega_s^{||} X^\perp\right] + \omega_s^\perp\right)\omega_t^T\right).$$

Both $X^\perp, \omega_s^{||}$ are projections, and since X follows an isotropic distribution, its right singular vectors (the eigenvectors of $X^\perp$) are Haar distributed. Now using Lemma 1 with $p = d - n_t, q = c_s$ we obtain that

$$\mathbb{E}_X\left[X^\perp \omega_s^{||} X^\perp\right] = \left(1 - \frac{n_t}{d}\right)\left[\frac{c_s n_t}{(d-1)(d+2)} I_{d\times d} + \left(1 - \frac{d n_t}{(d-1)(d+2)}\right)\omega_s^{||}\right].$$

Using $\omega_s^{||} = I_{d\times d} - \omega_s^\perp$ and reordering the terms we obtain

$$\mathbb{E}_X\left[X^\perp \omega_s^{||} X^\perp\right] + \omega_s^\perp = \left(1 - \frac{n_t}{d}\right)\left(1 - \frac{n_t(d-c_s)}{(d+2)(d-1)}\right) I_{d\times d}$$
$$+ \left(\frac{n_t}{d} + \frac{n_t(d-n_t)}{(d+2)(d-1)}\right)\omega_s^\perp.$$

Lastly, we use the standard result that $\mathcal{R}(\hat{\omega}_t) = \left(1 - \frac{n_t}{d}\right)$ (see e.g. [3]) and that $\varepsilon = \mathrm{Tr}\left(\omega_t \omega_s^\perp \omega_t^T\right)$ to conclude that

$$\mathcal{R}(\hat{\omega}_p \omega_s) = \left(1 - n_t \frac{d - c_s}{(d+2)(d-1)}\right)\mathcal{R}(\hat{\omega}_t) + n_t\left(\frac{1}{d} + \frac{d - n_t}{(d+2)(d-1)}\right)\varepsilon,$$

which completes the proof.

Using Lemma 1 and Theorem 3, we next prove Theorem 1, which is restated below for the reader's convenience.

Theorem. Let $\mathcal{X} = \mathbb{R}^d$, $\mathcal{Y}_s = \mathbb{R}^{c_s}$, $\mathcal{Y}_t = \mathbb{R}^{c_t}$, and let $\hat{\omega}_s = y_s X_s^\dagger$ and $\hat{\omega}_p = y_t(\hat{\omega}_s X_t)^\dagger$. Assuming that $\mathbb{P}_s$ and $\mathbb{P}_t$ are independent, isotropic distributions on $\mathbb{R}^d$, then the risk $\mathcal{R}(\hat{\omega}_p \hat{\omega}_s)$ is given by

$$\mathcal{R}(\hat{\omega}_p \hat{\omega}_s) = \left[(C_1 + C_2 K_1)\left(1 - \frac{n_t}{d}\right) + (1 - C_1 - C_2)\right]||\omega_t||_F^2 + C_2 K_2 \varepsilon,$$

where $\varepsilon = ||\omega_t(I_{d\times d} - \omega_s^\dagger \omega_s)||_F^2$ and

$$C_1 = \frac{n_s c_s (d - n_s)}{d(d-1)(d+2)}, \quad C_2 = \frac{n_s\left[d(n_s+1) - 2\right]}{d(d-1)(d+2)},$$
$$K_1 = 1 - \frac{n_t(d - c_s)}{(d-1)(d+2)}, \quad K_2 = \frac{n_t}{d} + \frac{n_t(d-n_t)}{(d-1)(d+2)}.$$

Proof. We let $X = X_t$ to simplify notation. We follow the proof Theorem 3, but now account for the expectation with respect to X_s. Namely,

$$\mathcal{R}(\hat{\omega}_p \hat{\omega}_s) = \mathrm{Tr}\left(\mathbb{E}_{X_s, X}\left[\omega_t\left(X^\perp \hat{\omega}_s^{||} X^\perp + \hat{\omega}_s^\perp\right)\omega_t^T\right]\right)$$
$$= \mathrm{Tr}\left(\omega_t\left(\mathbb{E}_{X_s, X}\left[X^\perp \hat{\omega}_s^{||} X^\perp\right] + \hat{\omega}_s^\perp\right)\omega_t^T\right).$$

Using the independence of $\hat{\omega}_s$ and $X^\perp$ and Fubini's theorem, we compute the expectations sequentially:

$$\mathcal{R}(\hat{\omega}_p \hat{\omega}_s) = \mathrm{Tr}\left(\omega_t\left(\mathbb{E}_X\left[X^\perp \mathbb{E}_{X_s}\left[\hat{\omega}_s^{||}\right] X^\perp\right] + \mathbb{E}_{X_s}\left[\hat{\omega}_s^\perp\right]\right)\omega_t^T\right).$$

Now, since $\hat{\omega}_s = \omega_s X_s^{||}$, we have that $\hat{\omega}_s^{\dagger} = X_s^{||}\omega_s^{\dagger}$. Therefore, $\hat{\omega}_s^{||} = \hat{\omega}_s^{\dagger}\hat{\omega}_s = X_s^{||}\omega_s^{||}X_s^{||}$. Similarly, $\hat{\omega}_s^{\perp} = I_{d\times d} - \hat{\omega}_s^{||}$. As a consequence, we calculate the two expectations involving X_s by using Lemma 1 with $p = n_s, q = c_s$. In particular, we conclude that

$$\begin{aligned}
\mathbb{E}_{X_s}\left[\hat{\omega}_s^{||}\right] &= C_1 I_{d\times d} + C_2\omega_s^{||} = (C_1 + C_2)I_{d\times d} - C_2\omega_s^{\perp},\\
\mathbb{E}_{X_s}\left[\hat{\omega}_s^{\perp}\right] &= (1 - C_1 - C_2)I_{d\times d} + C_2\omega_s^{\perp}.
\end{aligned}$$

Therefore, $\mathcal{R}(\hat{\omega}_p\hat{\omega}_s)$ is given by the sum of the following terms:

$$\begin{aligned}
C_1 \operatorname{Tr}\left(\omega_t \mathbb{E}_X\left[X^{\perp}\right]\omega_t^T\right) &= C_1\mathcal{R}(\hat{\omega}_t),\\
C_2 \operatorname{Tr}\left(\omega_t\left(\mathbb{E}_X\left[X^{\perp}\omega^{||}X^{\perp}\right]\right)\omega_t^T\right) &= C_2K_1\mathcal{R}(\hat{\omega}_t) + C_2(K_2 - 1)\varepsilon,\\
(1 - C_1 - C_2)\operatorname{Tr}\left(\omega_t\omega_t^T\right) + C_2\operatorname{Tr}\left(\omega_t\omega_s^{\perp}\omega_t^T\right) &= (1 - C_1 - C_2)\operatorname{Tr}\left(\omega_t\omega_t^T\right) + C_2\varepsilon, \ ,
\end{aligned}$$

where for the second equality, we applied Theorem 3, which gives rise to K_1 and K_2, thereby completing the proof.

3 Proof of Corollary 1

We restate Corollary 1 below for the reader's convenience.

Corollary. Let $S = \frac{n_s}{d}, T = \frac{n_t}{d}, C = \frac{c_s}{d}$ and assume $\|\omega_t\|_F = \Theta(1)$. Under the setting of Theorem 1, if $S, T, C < \infty$ as $d \to \infty$, then:

a) $\mathcal{R}(\hat{\omega}_p\hat{\omega}_s)$ is monotonically decreasing for $S \in [0, 1]$ if $\varepsilon < (1 - C)\|\omega_t\|_F$.

b) If $2S - 1 - ST < 0$, then $\mathcal{R}(\hat{\omega}_p\hat{\omega}_s)$ decreases as C increases.

c) If $S = 1$, then $\mathcal{R}(\hat{\omega}_p\hat{\omega}_s) = (1 - T + TC)\mathcal{R}(\hat{\omega}_t) + \varepsilon T(2 - T)$.

d) If $S = 1$ and $T, C = \Theta(\delta)$, then $\mathcal{R}(\hat{\omega}_p\hat{\omega}_s) = (1 - 2T)\|\omega_t\|_F^2 + 2T\varepsilon + \Theta(\delta^2)$.

Proof. We first derive forms for the terms C_1, C_2, K_1, K_2 from Theorem 1 as $d \to \infty$. In particular, we have:

$$C_1 = SC - S^2C \quad ; \quad C_2 = S^2 \quad ; \quad K_1 = 1 - T + TC \quad ; \quad K_2 = 2T - T^2.$$

Substituting these values into $\mathcal{R}(\hat{\omega}_p\hat{\omega}_s)$ in Theorem 1, we obtain

$$\mathcal{R}(\hat{\omega}_p\hat{\omega}_s) = \left[1 - 2S^2T + S^2T^2 + (2S - 1 - ST)STC\right]\|\omega_t\|_F^2 + S^2T[2 - T]\varepsilon. \tag{1}$$

Next, we analyze Eq. (1) for $S \in [0, 1]$. For fixed T and C, it holds that $\mathcal{R}(\hat{\omega}_p\hat{\omega}_s)$ is a quadratic in S and given by

$$\mathcal{R}(\hat{\omega}_p\hat{\omega}_s) = (1 - STC)\|\omega_t\|_F^2 - S^2T[2 - T][1 - C]\|\omega_t\|_F^2 + S^2T[2 - T]\varepsilon.$$

For $S \in [0, 1]$ and $\varepsilon < (1 - C)\|\omega_t\|_F^2$, this quadratic is strictly decreasing and thus we can conclude that $\mathcal{R}(\hat{\omega}_p\hat{\omega}_s)$ is decreasing in S. We next observe that $\mathcal{R}(\hat{\omega}_p\hat{\omega}_s)$ is linear in C and thus $\mathcal{R}(\hat{\omega}_p\hat{\omega}_s)$ decreases as C increases if and only if the coefficient of C is negative, i.e. $(2S - ST - 1) < 0$. Lastly, if $S = 1$, then

$$\begin{aligned}
\mathcal{R}(\hat{\omega}_p\hat{\omega}_s) &= [(1 - T)^2 + (1 - T)TC]\|\omega_t\|_F^2 + T[2 - T]\varepsilon\\
&= (1 - T)(1 - T + TC)\|\omega_t\|_F^2 + T[2 - T]\varepsilon\\
&= (1 - T + TC)\mathcal{R}(\hat{\omega}_t) + T[2 - T]\varepsilon.
\end{aligned}$$

Corollary 1c, d follow from the above form of the risk, thus completing the proof.

4 Equivalence of Fine-tuned and Translated Linear Models

We now prove that for linear models transfer learning using the translated predictor from Definition 2 is equivalent to transfer learning via the conventional fine-tuning process. This follows from Proposition 1 below, which implies that when parameterized by a linear model, the translated predictor is the interpolating solution for the target dataset that is nearest to the source predictor.

Proposition 1. Let $\hat{f}_s(x) = \langle w_s, \psi(x)\rangle_{\mathcal{H}}$, where $\psi : \mathbb{R}^d \to \mathcal{H}$ is a feature map and $\mathcal{H}$ is a Hilbert space. Then the translated predictor, $\hat{f}_t$, is the solution to

$$\begin{aligned} &\arg\min_{w} \|w - w_s\|_{\mathcal{H}} \\ &\text{subject to } \langle w, \psi(X_t)\rangle_{\mathcal{H}} = y_t. \end{aligned} \tag{2}$$

Proof. Note that any solution w to Problem 2 can be written as $w = w_s + \tilde{w}$. Hence, we can rewrite Problem 2 as follows:

$$\begin{aligned} &\arg\min_{\tilde{w}} \|\tilde{w}\|_{\mathcal{H}} \\ &\text{subject to } \langle w_s + \tilde{w}, \psi(X_t)\rangle_{\mathcal{H}} = y_t, \end{aligned}$$

where the constraint can be simplified to $\langle \tilde{w}, \psi(X_t)\rangle_{\mathcal{H}} = y_t - \hat{f}_s(X_t)$. This is precisely the constraint for the translated predictor in Definition 2, thereby completing the proof.

5 Proof of Theorem 2

We restate Theorem 2 below for convenience and then provide the proof.

Theorem. Let $\mathcal{X} = \mathbb{R}^d$, $\mathcal{Y}_s = \mathbb{R}^{c_s}$, $\mathcal{Y}_t = \mathbb{R}^{c_t}$, and let $\hat{\omega}_t = \hat{\omega}_s + \hat{\omega}_c$ where $\hat{\omega}_s = y_s X_s^{\dagger}$ and $\hat{\omega}_c = (y_t - \hat{\omega}_s X_t)X_t^{\dagger}$. Assuming that $\mathbb{P}_s$ and $\mathbb{P}_t$ are independent, isotropic distributions on $\mathbb{R}^d$, the the risk $\mathcal{R}(\hat{\omega}_t)$ is given by

$$\mathcal{R}(\hat{\omega}_t) = \left[\frac{\|\omega_s - \omega_t\|_F^2}{\|\omega_t\|_F^2} + \left(1 - \frac{n_s}{d}\right)\left(1 - \frac{\|\omega_s - \omega_t\|_F^2}{\|\omega_t\|_F^2}\right)\right]\mathcal{R}(\hat{\omega}_b),$$

where $\hat{\omega}_b = y_t X_t^{\dagger}$ is the baseline predictor.

Proof. We prove the statement by directly simplifying the risk as follows.

$$\begin{aligned}
\mathcal{R}(\hat{\omega}_t) &= \mathbb{E}_{x,X_s,X_t}\left[(\hat{\omega}_t x - \omega_t x)^2\right] \\
&= \mathbb{E}_{X_s,X_t}\left[\|\hat{\omega}_t - \omega_t\|_F^2\right] \\
&= \mathbb{E}_{X_s,X_t}\left[\|\hat{\omega}_s + (y_t - \hat{\omega}_s X_t)X_t^{\dagger} - \omega_t\|_F^2\right] \quad \text{(By Definition 2)} \\
&= \mathbb{E}_{X_s,X_t}\left[\|\hat{\omega}_s(I - X_t X_t^{\dagger}) - \omega_t(I - X_t X_t^{\dagger})\|_F^2\right] \quad (\text{As } y_t = \omega_t X_t) \\
&= \left(1 - \frac{n_t}{d}\right)\mathbb{E}_{X_s}\left[\|\hat{\omega}_s - \omega_t\|_F^2\right] \quad \left(\text{As } \mathbb{E}_{X_t}[X_t X_t^{\dagger}] = \frac{n_t}{d}\right) \\
&= \left(1 - \frac{n_t}{d}\right)\mathbb{E}_{X_s}\left[\|\omega_s X_s X_s^{\dagger}\|_F^2 + \|\omega_t\|_F^2 - 2\langle \omega_s X_s X_s^{\dagger}, \omega_t\rangle\right] \\
&= \left(1 - \frac{n_t}{d}\right)\left[\frac{n_s}{d}\|\omega_s\|_F^2 + \|\omega_t\|_F^2 - \frac{2n_s}{d}\langle \omega_s, \omega_t\rangle\right] \\
&= \left(1 - \frac{n_t}{d}\right)\left[\|\omega_s - \omega_t\|_F^2 + \left(1 - \frac{n_s}{d}\right)\left(\|\omega_t\|_F^2 - \|\omega_t - \omega_s\|_F^2\right)\right] \\
&= \left[\frac{\|\omega_s - \omega_t\|_F^2}{\|\omega_t\|_F^2} + \left(1 - \frac{n_s}{d}\right)\left(1 - \frac{\|\omega_s - \omega_t\|_F^2}{\|\omega_t\|_F^2}\right)\right]\mathcal{R}(\hat{\omega}_b),
\end{aligned}$$

where the penultimate equality follows from adding and subtracting the term $\frac{n_s}{d}\|\omega_t\|_F^2$ and the last equality is given by $\mathcal{R}(\hat{\omega_b}) = \left(1 - \frac{n_t}{d}\right)\|w_t\|_F^2$, thereby completing the proof.

In the following, we extend Theorem 2 to kernel regression with nonlinear kernels under the assumption that the true target function is linear in the Hilbert space under the given kernel feature map.

Theorem 4. Let $\mathcal{X} = \mathbb{R}^d$, $\mathcal{Y}_s = \mathcal{Y}_t = \mathbb{R}$, let $\mathcal{H}$ be a Hilbert space, and let $\psi : \mathcal{X} \to \mathcal{H}$ induce a positive semi-definite, bounded kernel $K(x, z) = \langle \psi(x), \psi(z)\rangle$. Let $f_s = \psi(X_s)\alpha_s$ denote the source model corresponding to solving kernel regression on source data $(X_s, y_s) \in \mathcal{X}^{d\times n} \times \mathcal{Y}_s^{c\times n}$ with $\psi(X_s) = \left[\psi(x_s^{(1)})|\psi(x_s^{(2)})|\ldots|\psi(x_s^{(n)})\right]$. Assume the oracle target function, f^*, has the form $f^*(x) = \langle w^*, \psi(x)\rangle_{\mathcal{H}}$.

Let $\hat{f}$ denote the solution to kernel regression on the target data and let $\hat{f}_t$ denote the translated solution. Then for target data $(X_t, y_t) \in \mathbb{R}^{d\times n} \times \mathbb{R}^{c\times n}$, it holds that

$$\mathcal{R}(\hat{f}) \leq \mathbb{E}_{X_t}[\|(I-P)(w^*)\|_{\mathcal{H}}^2]\lambda,$$
$$\mathcal{R}(\hat{f}_t) \leq \mathbb{E}_{X_t}[\|(I-P)(w^* - \psi(X_s^T)\alpha_s)\|_{\mathcal{H}}^2]\lambda,$$

where $P = \psi(X_t)(\psi(X_t)^T\psi(X_t))^{-1}\psi(X_t)^T$ is a self-adjoint, compact operator and $\lambda = \mathbb{E}_x[K(x,x)]$.

Proof. We will prove the statement for the translated predictor since the corresponding statement for the base predictor follows immediately from $f_s = 0$. The translated predictor $\hat{f}_t$ can be written as

$$\hat{f}_t(x) = K(x, X_t)K_t^{-1}y_t^T - K(x, X_t)K_t^{-1}f_s(X_t) + f_s(x) = \psi(x^T)\beta,$$

for $\beta = \psi(X_t)K_t^{-1}y_t^T - \psi(X_t)K_t^{-1}f_s(X_t) + \psi(X_s)\alpha_s \in \mathcal{H}$. Thus, the risk of this predictor is given by

$$\begin{aligned}
\mathcal{R}(\hat{f}_t) &= \mathbb{E}_{x,X_t}\left[(f^*(x) - \hat{f}_t(x))^2\right] \\
&= \mathbb{E}_{x,X_t}\left[(\langle\psi(x), w^*\rangle - \langle\psi(x), \beta\rangle)^2\right] \\
&= \mathbb{E}_{x,X_t}\left[(\langle\psi(x), w^* - \beta\rangle)^2\right] \\
&\leq \mathbb{E}_{x,X_t}\left[\|w^* - \beta\|^2\|\psi(x)\|^2\right] \qquad \text{(by Cauchy-Schwarz)} \\
&= \mathbb{E}_{x,X_t}\left[\|w^* - \beta\|^2 K(x,x)\right] \\
&= \lambda\mathbb{E}_{X_t}\left[\|w^* - \beta\|^2\right].
\end{aligned}$$

Now it remains to be shown that $(w^* - \beta) = (I - P)(w^* - \psi(X_s)^T\alpha_s)$. We simplify $w^* - \beta$ directly as follows:

$$\begin{aligned}
w^* - \beta &= w^* - \psi(X_t)K_t^{-1}y_t^T + \psi(X_t)K_t^{-1}f_s(X_t) - \psi(X_s)\alpha_s \\
&= w^* - \psi(X_t)K_t^{-1}\psi(X_t^T)w^* + \psi(X_t)K_t^{-1}\psi(X_t^T)\psi(X_s)\alpha_s - \psi(X_s)\alpha_s \\
&= (I-P)w^* - (I-P)\psi(X_s)\alpha_s \\
&= (I-P)(w^* - \psi(X_s)\alpha_s),
\end{aligned}$$

which concludes the proof.

Theorem 4 provides a natural extension to Theorem 2 since it shows that the risk of the translated predictor is reduced when the parameters of the source function, $\psi(X_s)\alpha_s$, and those of the true target function, w^*, are close in norm.

Supplementary Figures

(a)

	CIFAR10		Flowers102		DTD		SVHN (500 target ex.)	
CNN	60.32%	**75.85%**	15.79%	**34.77%**	10.69%	**23.30%**	38.62%	**55.31%**
CNN Early Stopped	60.64%	**68.53%**	15.61%	**28.80%**	10.74%	**18.51%**	38.63%	**46.33%**

☐ Baseline Predictor ☐ Predictor Transferred from ImageNet32

(b) CNN (CIFAR10 to CIFAR-C)

Corruption	Source Acc.	Baseline Acc.	Transferred Acc.
Brightness	52.50%	49.50%	**58.60%**
Contrast	23.90%	39.20%	**56.10%**
Defocus Blur	55.20%	50.70%	**59.50%**
Elastic Transform	55.80%	49.80%	**58.00%**
Fog	27.30%	40.80%	**48.90%**
Frost	43.80%	46.50%	**56.30%**
Gaussian Blur	53.70%	52.20%	**59.10%**
Gaussian Noise	49.70%	46.90%	**56.70%**
Glass Blur	52.10%	48.40%	**54.90%**
Impulse Noise	37.70%	45.40%	**53.50%**
Jpeg Compression	60.00%	50.70%	**59.80%**
Motion Blur	49.80%	49.50%	**56.70%**
Pixelate	58.40%	48.70%	**60.00%**
Saturate	48.90%	51.40%	**58.80%**
Shot Noise	50.60%	46.00%	**57.50%**
Snow	50.70%	46.30%	**54.30%**
Spatter	54.40%	48.70%	**56.60%**
Speckle Noise	49.60%	47.60%	**55.50%**
Zoom Blur	53.20%	49.80%	**58.60%**
Average	48.81%	47.79%	**56.81%**

(c) CNN (ImageNet32 to CIFAR-C)

Corruption	Baseline Acc.	Transferred Acc.
Brightness	50.80%	**67.90%**
Contrast	39.80%	**66.00%**
Defocus Blur	52.10%	**67.20%**
Elastic Transform	47.30%	**65.10%**
Fog	40.00%	**64.20%**
Frost	46.10%	**62.40%**
Gaussian Blur	50.50%	**64.90%**
Gaussian Noise	46.00%	**62.60%**
Glass Blur	50.00%	**63.10%**
Impulse Noise	44.80%	**52.20%**
Jpeg Compression	49.10%	**66.50%**
Motion Blur	49.90%	**65.60%**
Pixelate	51.20%	**67.20%**
Saturate	53.20%	**67.40%**
Shot Noise	46.00%	**61.80%**
Snow	47.50%	**63.90%**
Spatter	47.20%	**65.00%**
Speckle Noise	47.00%	**60.80%**
Zoom Blur	49.60%	**66.90%**
Average	47.79%	**64.25%**

Supplementary Fig. S1: Image classification performance of CNNs that are finite-width analogs of the CNTK considered in this work. (a) The accuracy of the CNNs on 4 target tasks when transferred from ImageNet32. All layers of the CNNs are fine-tuned during transfer learning. The CNN in the top row achieves a test accuracy of 16.72% on ImageNet32. The early stopped CNN in the bottom row achieves an accuracy of 10.692% on ImageNet32, which is comparable with the accuracy of the CNTK (10.64%). (b) Performance of a CNN pre-trained on CIFAR10 when transferred to CIFAR-C. (c) Performance of a CNN pre-trained on ImageNet32 when transfered to CIFAR-C.

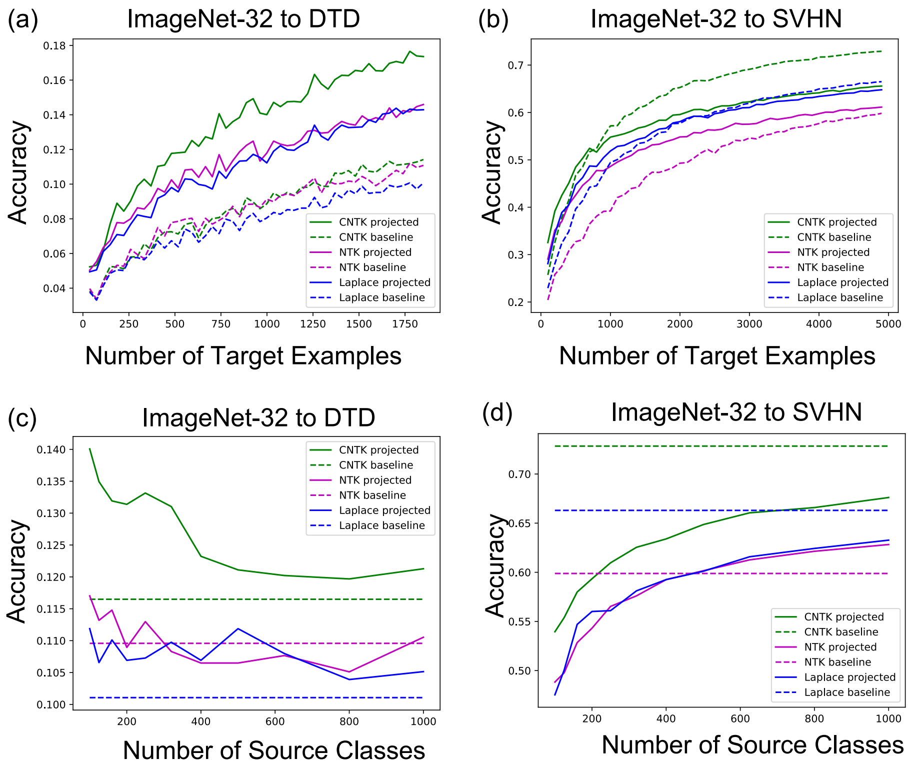

Supplementary Fig. S2: (a, b) Performance of the projected kernel method as a function of the number of target examples when transferred from ImageNet32 to DTD and SVHN. (c, d) Performance of the projected kernel method as a function of the number of source classes when transferred from ImageNet32 to DTD and SVHN. The number of source examples was fixed to 40k and we ensured that the number of source classes divides 40k.

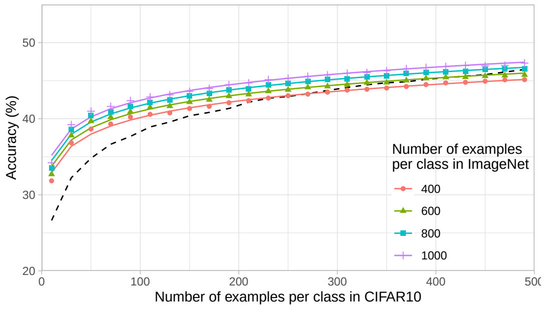

(a) Laplace Kernel

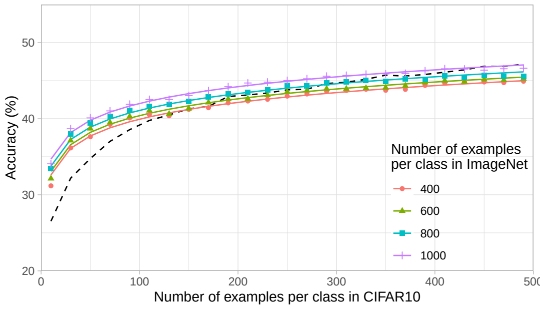

(b) NTK

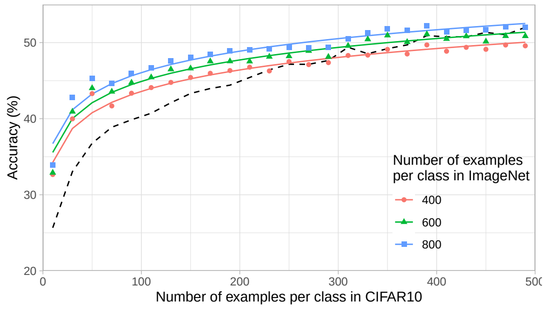

(c) CNTK

Supplementary Fig. S3: Performance of (a) Laplace kernel, (b) Neural Tangent Kernel (NTK), and (c) Convolutional Neural Tangent Kernel (CNTK) as a function of the number of source examples and target examples when projected from ImageNet32 to CIFAR10. The baseline predictor performance is shown as a dashed black line. Overall, we find that performance improves as the number of source training samples per class increases.

	CNTK	NTK	Laplace
CIFAR10	0.996	0.997	0.998
Flowers	0.951	0.953	0.953
DTD	0.965	0.962	0.944
SVHN	0.990	0.996	0.993

Supplementary Fig. S4: R^2 values given by fitting the coefficients a, b of the curve $y = a \log_2 x + b$ to the curves found empirically for the projected predictor performance as a function of the target samples. We see that for all kernels and datasets, the fit yields R^2 values greater than 0.94 and values higher than 0.99 on datasets with more samples such as CIFAR10 and SVHN.

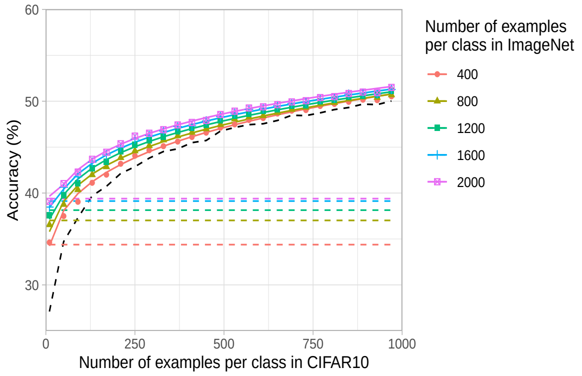

(a) Laplace Kernel

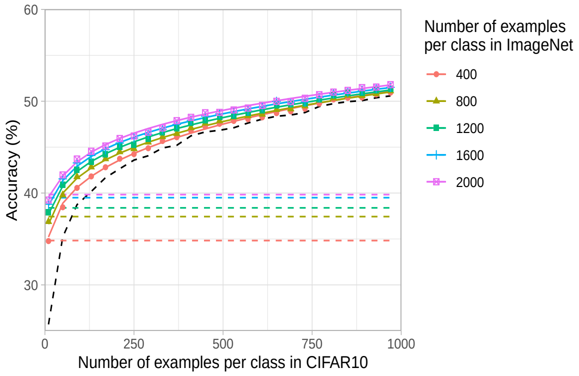

(b) Neural Tangent Kernel

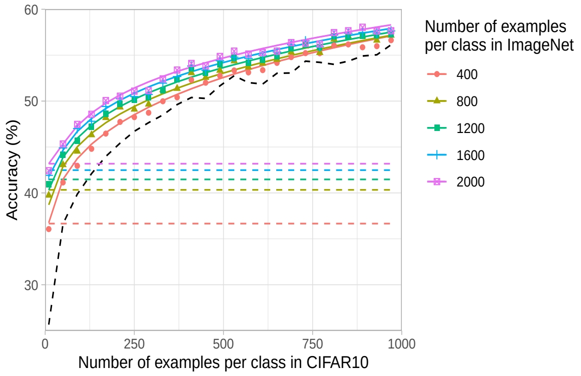

(c) Convolutional Neural Tangent Kernel

Supplementary Fig. S5: Accuracy of the translated predictor from ImageNet32 to CIFAR10 when using the following predictors: (a) Laplace kernel ; (b) Neural Tangent Kernel ; and (c) Convolutional Neural Tangent Kernel. The black dashed line corresponds to the baseline predictor while the dashed color lines correspond to the source predictors. We observe that the translated predictor outperforms both projected and baseline predictors when increasing the number of target samples, and the performance of the translated predictor increases as the number of source examples per class increases.

CNTK

Corruption	Source Acc.	Baseline Acc.	Projected Acc.	Translated Acc.
Brightness	60.80%	54.60%	60.00%	**65.80%**
Contrast	41.60%	50.00%	46.50%	**59.80%**
Defocus Blur	63.30%	54.80%	61.90%	**65.00%**
Elastic Transform	63.70%	54.30%	62.00%	**64.00%**
Fog	32.70%	43.00%	37.80%	**49.00%**
Frost	57.90%	53.90%	59.60%	**62.70%**
Gaussian Blur	62.40%	55.00%	61.10%	**64.90%**
Gaussian Noise	53.10%	48.20%	59.30%	**61.90%**
Glass Blur	62.20%	52.50%	60.80%	**63.60%**
Impulse Noise	42.00%	46.80%	55.20%	**57.20%**
Jpeg Compression	66.20%	54.90%	64.20%	**67.10%**
Motion Blur	60.10%	55.40%	60.30%	**63.20%**
Pixelate	65.30%	57.00%	63.90%	**66.70%**
Saturate	55.70%	57.00%	58.10%	**65.20%**
Shot Noise	52.60%	49.00%	59.60%	**60.80%**
Snow	59.60%	50.40%	57.70%	**61.80%**
Spatter	61.60%	52.70%	62.50%	**65.00%**
Speckle Noise	53.00%	49.60%	59.20%	**62.90%**
Zoom Blur	61.60%	55.60%	62.40%	**65.20%**
Average	56.60%	52.35%	58.53%	**62.73%**

NTK

Corruption	Source Acc.	Baseline Acc.	Projected Acc.	Translated Acc.
Brightness	51.30%	46.80%	48.90%	**54.70%**
Contrast	36.90%	44.40%	44.60%	**51.40%**
Defocus Blur	54.00%	47.80%	51.00%	**55.10%**
Elastic Transform	53.50%	45.10%	49.10%	**54.10%**
Fog	28.60%	35.10%	28.80%	**41.30%**
Frost	48.00%	42.10%	49.10%	**50.80%**
Gaussian Blur	53.80%	47.10%	51.60%	**54.80%**
Gaussian Noise	52.50%	43.70%	50.70%	**52.70%**
Glass Blur	52.70%	45.60%	51.60%	**52.90%**
Impulse Noise	50.80%	41.40%	50.00%	**50.40%**
Jpeg Compression	54.30%	45.10%	51.40%	**54.10%**
Motion Blur	52.70%	44.90%	49.20%	**53.80%**
Pixelate	55.90%	45.90%	51.00%	**55.50%**
Saturate	50.00%	50.40%	50.60%	**57.30%**
Shot Noise	53.50%	43.50%	50.80%	**53.40%**
Snow	48.30%	42.50%	48.70%	**49.40%**
Spatter	51.60%	44.20%	49.10%	**53.30%**
Speckle Noise	52.80%	43.50%	50.40%	**52.60%**
Zoom Blur	52.90%	45.10%	49.80%	**54.90%**
Average	50.22%	44.43%	48.76%	**52.76%**

Laplace Kernel

Corruption	Source Acc.	Baseline Acc.	Projected Acc.	Translated Acc.
Brightness	48.00%	48.60%	46.80%	**54.90%**
Contrast	38.70%	48.90%	43.10%	**53.30%**
Defocus Blur	58.70%	49.30%	56.00%	**58.50%**
Elastic Transform	58.00%	48.70%	54.20%	**58.10%**
Fog	26.50%	33.70%	28.20%	**40.90%**
Frost	49.90%	44.80%	50.70%	**54.10%**
Gaussian Blur	57.90%	49.20%	56.30%	**57.80%**
Gaussian Noise	56.10%	46.90%	54.50%	**56.30%**
Glass Blur	56.40%	46.90%	55.60%	**57.10%**
Impulse Noise	52.70%	43.40%	52.50%	**54.10%**
Jpeg Compression	58.60%	49.00%	54.60%	**58.20%**
Motion Blur	56.10%	48.50%	52.80%	**59.40%**
Pixelate	59.70%	49.00%	54.40%	**58.50%**
Saturate	51.60%	51.90%	50.30%	**60.40%**
Shot Noise	56.30%	46.60%	53.20%	**57.10%**
Snow	45.30%	44.80%	45.40%	**52.10%**
Spatter	55.70%	46.70%	54.10%	**57.50%**
Speckle Noise	55.10%	46.40%	53.70%	**56.30%**
Zoom Blur	56.60%	48.40%	52.20%	**58.30%**
Average	52.52%	46.93%	50.98%	**55.94%**

Supplementary Fig. S6: Performance of kernels translated from CIFAR10 to CIFAR-C. We observe that for all 19 perturbations, the translated predictor outperforms the source, baseline, and projected predictors.

(a)

	CKA Source Model Accuracy: 93.55%		
	CKA Baseline	CKA Transfer	CKA Translation
Average Acc. over 19 Corruptions	84.92%	87.05%	**92.01%**

(b)

	EasyMKL Source Model Accuracy: 91.95%		
	EasyMKL Baseline	EasyMKL Transfer	EasyMKL Translation
Average Acc. over 19 Corruptions	65.29%	76.79%	**87.55%**

(c)

	FHeuristic Source Model Accuracy: 91.65%		
	FHeuristic Baseline	FHeuristic Transfer	FHeuristic Translation
Average Acc. over 19 Corruptions	75.36%	73.81%	**85.19%**

(d)

	PWMK Source Model Accuracy: 91.50%		
	PWMK Baseline	PWMK Transfer	PWMK Translation
Average Acc. over 19 Corruptions	69.83%	70.24%	**81.53%**

Supplementary Fig. S7: Comparison between (1) the baseline model in which we use the kernel learning algorithm directly on the target task ; (2) the transferred learned kernel model in which we use the weights for combining the kernels on the source task to combine the kernel on the target task prior to solving kernel regression ; and (3) translating the learned kernel on the source task using our translation methodology. We train a source model on the binary classification task of cars vs. deer in CIFAR10 (10000 training examples) and compare average test target accuracy when transferring to cars vs. deer classification under 19 corruptions from CIFAR10-C (1000 training, 1000 test examples). We use multiple kernel learning to combine the Laplace kernel, Gaussian kernel and linear kernel and compare with four multiple kernel learning algorithms implemented in [4]: (a) Centered Kernel Alignment (CKA); (b) EasyMKL ; (c) FHeuristic ; and (d) Proportionally Weighted Multiple Kernels (PWMK).

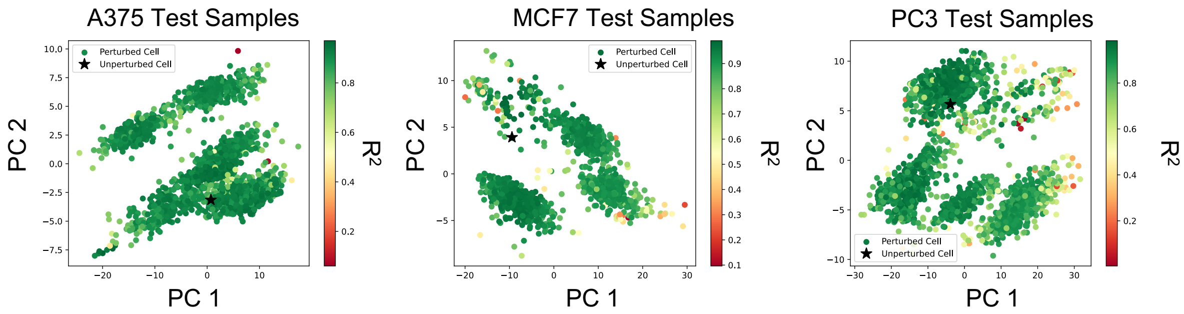

Supplementary Fig. S8: Performance (in R^2) of the transferred kernels with respect to the first two principal components (denoted PC1 and PC2) of gene expression for the A375, MCF7, and PC3 cell lines.

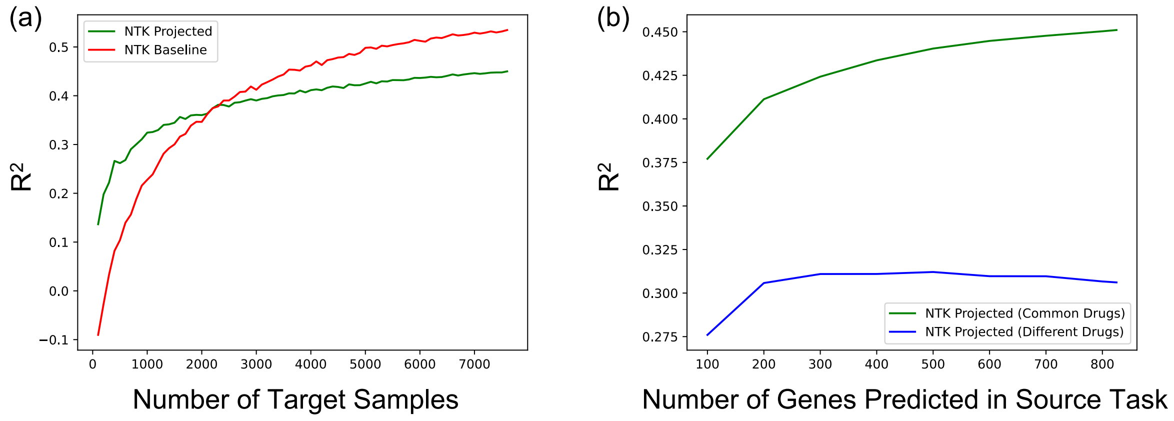

Supplementary Fig. S9: Analysis of projecting the kernel predictors of gene expression in CMAP to viability score in DepMap. (a) We observe up to a 0.2 boost in R^2 values when projecting to new cell lines for which the drugs were available in the source task. (b) We observe that predicting more genes in the source task is helpful when transferring to new cell lines for which the considered drugs were available in the source task. Predicting more genes for new cell lines for which the considered drugs were not available in the source task is harmful when transfer learning.

Supplementary References

1. Schölkopf, B. & Smola, A. J. *Learning with Kernels: Support Vector Machines, Regularization, Optimization, and Beyond.* (MIT Press, 2002).
2. Chatterjee, S. & Meckes, E. Multivariate normal approximation using exchangeable pairs. *ALEA Lat. Am. J. Probab. Math. Stat.* **4** (Jan. 2007).
3. Belkin, M., Hsu, D. & Xu, J. Two models of double descent for weak features. *Society for Industrial and Applied Mathematics Journal on Mathematics of Data Science* **2,** 1167–1180 (2020).
4. Lauriola, I. & Aiolli, F. MKLpy: a python-based framework for Multiple Kernel Learning. *arXiv preprint arXiv:2007.09982* (2020).
